# Rethinking Database Architectures for Memory-Centric Computing

**DOI:** 10.1007/s13222-026-00556-6

**Published:** 2026-08-24

**Authors:** Michail Georgoulakis Misegiannis, Abdelrahman Adel, Mateusz Gienieczko, Jana Giceva

**Affiliations:** Technical University of Munich, Munich, Germany

**Keywords:** Disaggregated Memory, CXL, Database Systems, Memory-Centric Computing

## Abstract

Datacenter architectures have steadily moved toward resource disaggregation, storage first, and now memory itself. This shift is arriving through several competing fabrics (RDMA, CXL pooling) that show no sign of converging.

Their shared features lead to treating them as one class: memory has stopped being local, uniform, or CPU-owned. But their differences in latency, coherence, ownership, and failure behavior are large enough that a database engine wired to any one of them is costly to retarget when the next one arrives.

In this position paper, we present a memory-centric design model in which a database expresses what it needs from memory as declarative properties and a runtime resolves them against whichever fabric is present. We consolidate three architectural principles from recent work that instantiate this approach at different layers of the stack: logical memory regions, declarative memory services, and state-centric task DAGs.

## Introduction

For most of the history of database systems, memory has been a property of a single-node server: locally attached, cache-coherent DRAM whose latency, bandwidth, and capacity were determined by the socket closest to it. The core components of data processing systems—buffer managers, query engines, and transaction logs, among others—have all been co-designed against that assumption for decades. At hyperscaler scale, however, this assumption has become a major source of inefficiency: memory now dominates rack-scale financial and energy budgets, yet much of it sits underutilized [10].

In today’s cloud, compute and memory are provisioned together as a unit, leaving memory routinely subject to *resource stranding*: it sits unused on one instance while tenants on others are starved for it. At Azure, for example, roughly half of all virtual machines use less than half of the memory they pay for, leaving fleet-wide capacity 35–50% idle [20].

**Fig. 1:**
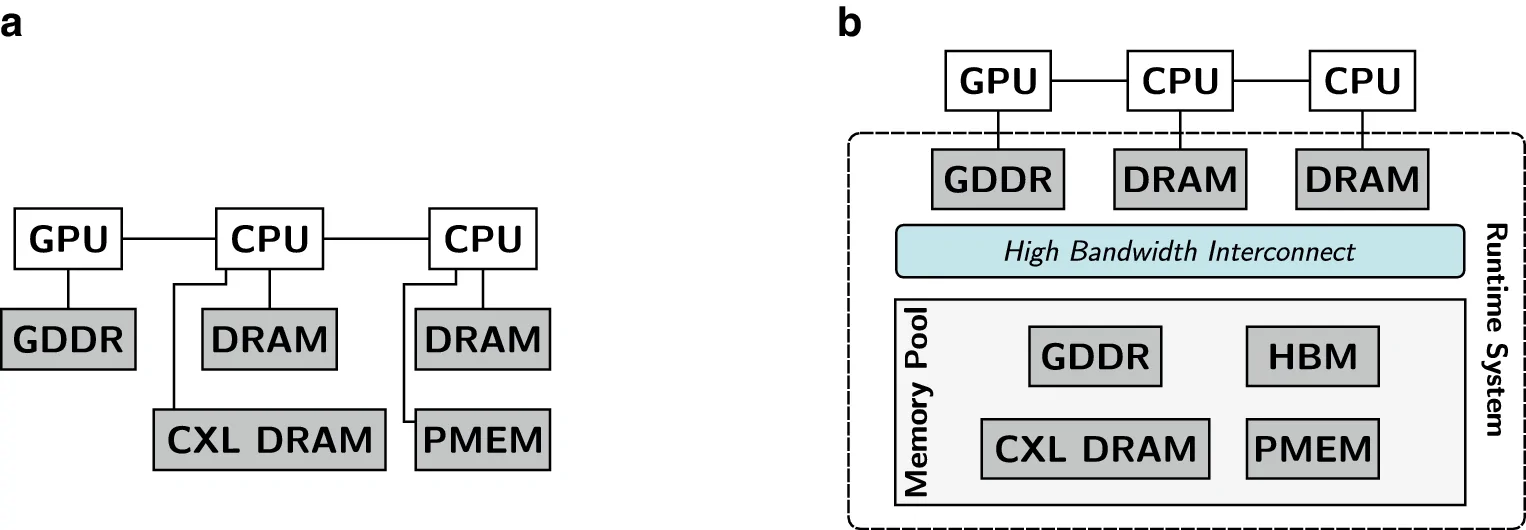
Current processor-centric architectures vs. future memory-centric architectures where memory is managed by a runtime. The runtime transparently handles data placement and movement. **a** Current architectures. **b** Our vision for memory-centric systems

Closing this gap is the next step in a longer disaggregation trend. The same architectural move that earlier decoupled storage from compute now applies to memory: decoupling it from the CPU socket and pooling it across the fabric, so that memory itself becomes a primary unit around which datacenters are organized (Fig. 1).

This disaggregation is being realized through several distinct hardware technologies that target different parts of the problem.

- *RDMA* over InfiniBand or modern Ethernet has long been in production and underpins distributed-database designs built on shared-memory abstractions over fast networks [27, 31]; its accesses, however, remain explicit, message-based, and non-coherent.
- *Compute Express Link* (CXL) extends the local memory hierarchy across servers with cache-coherent, byte-addressable accesses at near-DRAM latencies [25, 26], and has emerged in several flavors: single-server memory-expansion cards [18], switched pools across a pod [20], and switchless multi-ported devices in sparser topologies [30].
- *Emerging links* extend coherent shared memory across new boundaries: NVLink and in-package System-on-Chip designs unify accelerator and host memory in a single cache-coherent address space [7, 9], blurring the boundary between host and accelerator memory; photonic interconnects promise to extend coherent domains beyond the rack, though they remain largely research prototypes [23].

These fabrics differ substantially in latency, bandwidth, coherence semantics, and access granularity, and the differences are large enough to propagate up the stack to the application. A data processing system that treats every substrate the same fails to extract the performance each is capable of. One tuned to a specific fabric performs well as long as that technology remains dominant; once the landscape shifts, adapting the engine requires invasive rewrites. Both strategies fail symmetrically: uniform treatment trades the gains each fabric offers for portability, while fine-tuning trades portability for an invasive rewrite at every hardware shift.

We argue that the right response to fragmentation is not to bet on a winning fabric but to abstract over it. We propose a set of memory-centric, technology-agnostic, declarative abstractions through which a database describes what it needs from memory and a runtime resolves those descriptions against whatever fabric is available. This is the direction our group at TUM has been pursuing in recent years, in collaboration with others in the community; the purpose of this article is to draw the resulting design together as a coherent program.

In this article, we make four contributions:

1. frame the memory-disaggregation landscape as a permanent condition of fragmentation across competing fabrics, and situate it against the broader literature;
2. propose a memory-centric design model that reorganizes the engine around the memory needs of individual tasks;
3. consolidate three architectural principles from recent work at our group—logical memory regions [4], declarative memory services [5], and annotated state-centric task DAGs [13];
4. outline the open research agenda this program raises—programming abstractions, runtime support, and evaluation.

## Memory-Centric Designs

Computer architectures are traditionally organized around the CPU. This convention extends to cloud-based deployments, which reproduce the model by sizing and billing instances by vCPUs and bundling memory with the vCPU count rather than provisioning it separately. As a result, data-processing systems built on these architectures inherit the same view, treating memory as a single uniform pool whose properties are those of the locally attached DRAM.

Traditional single-node query engines, for instance, read and write through a single shared address space, oblivious to the variety of the underlying hardware. This view does not survive, however, once memory becomes a pooled substrate, spread across tiers, and only partially coherent.

Memory disaggregation responds to this mismatch by inverting the inherited organization. Memory is now the substrate—a pooled resource addressable by any compute device—and the CPU socket becomes one of several compute peers around it. Compute, in turn, attaches heterogeneously: a single job may span a host CPU running the control flow and operators with heavy branching; a GPU over NVLink handling vectorizable work with an order of magnitude more memory bandwidth; a near-memory accelerator over CXL applying filters and projections without paying the round trip back to the host; and a remote pool over RDMA holding the cold spill or the cross-task shared state that does not fit locally. For a database engine, the central design question therefore shifts from extracting performance from a fixed local pool to expressing its needs to a substrate that reconfigures underneath it.

The database community has confronted a related question before, under the framing of the shared-disk versus shared-nothing debate, and more recently as a revived interest in shared-memory architectures over fast interconnects [18, 27]. These distinctions still capture meaningful differences in how engines structure compute and data placement. What has changed is the memory underneath: it has become heterogeneous, a graded substrate with several tiers that differ in latency, coherence, and persistence, where different tasks within the same job can need different sharing semantics from the same underlying fabric.

The relevant question thus becomes more fine-grained. For every piece of state (a buffer pool page, a hash-join build side, a transaction log tail), the engine has to know which tasks must see it, with what coherence and ordering guarantees, and over what lifetime; two objects in the same job may answer these questions differently.

This reframing motivates a specific kind of abstraction, one built to outlast the hardware it runs on. If the properties of the state matter, then the natural interface between the database and the memory fabric is one in which the database declares the properties it needs, and the runtime resolves those declarations against the available hardware. This mirrors how abstractions such as the buffer manager and virtual memory already hide physical data placement: an engine addresses a logical page, or an application a virtual address, while the (operating) system decides which pages are resident in fast memory and which are not.

The payoff is the same: the engine is written once, the runtime can choose different physical realizations, and the choice can change as the hardware changes without rewriting the engine. The declarations are not free-form; they range over a small, well-defined vocabulary of memory properties, which we adopt from our recent work [4, 5] and which Sect. 3 examines in detail.

The design model is future-proof in a narrow but useful sense: it confines the technology-specific concerns to one component, the runtime that resolves declarations to mechanisms, rather than spreading them through the buffer manager, the operators, and the transaction layer. When a new fabric arrives, only the resolver changes; the engine’s operators, task graphs, and property declarations do not. This stands in contrast to the prevailing pattern, in which a database fine-tuned for one fabric must be largely rewritten for the next.

The remainder of this article articulates this design model through three architectural principles drawn from recent work [4, 5, 13], each of which instantiates the same declarative, property-driven approach at a different layer of the stack.

## Architectural Principles

The vision we outline rests on *three architectural principles*, each at a layer where current systems hard-code assumptions about memory. At every level of today’s data stack, device choices are made statically at design time: the application allocates on a specific heap, the engine wires the access path into the operator, and the runtime treats remote and persistent memory as I/O behind the buffer pool.

The first principle concerns the application’s view of memory: programs should describe what they need from a memory region rather than which device should supply it. The second concerns the data processing engine itself: its operators and task DAGs should expose enough structure for the runtime to make placement and scheduling decisions, rather than coupling hardware assumptions into operator implementations. The third concerns the runtime: once applications declare their requirements and the engine exposes its workload, the runtime should orchestrate caching, tiering, replication, and offloading as services that satisfy those declarations against whatever hardware is present.

Taken together, the three principles describe a single move: pushing hardware-specific decisions out of application code and operators, and into a runtime that mediates between declarative requirements and a heterogeneous memory fabric.

### Logical Memory Regions

#### The Coupling Problem

The first principle is that memory should be declared by the properties an application needs rather than by the device that supplies it. Today’s programming models still assume a flat, locally attached address space: the developer allocates on a specific device, and the resulting code is implicitly tied to the latency, bandwidth, granularity, coherence, and persistence of that device. Once any of those parameters change, whether because the workload is moved to a node with CXL-attached DRAM, because part of the working set is offloaded to a GPU, or because data is shared with a remote peer over RDMA, the placement decisions baked into the code stop being optimal, and, when a new device offers different guarantees than the code assumed, stop being correct.

#### Property-Defined Regions

**Fig. 2:**
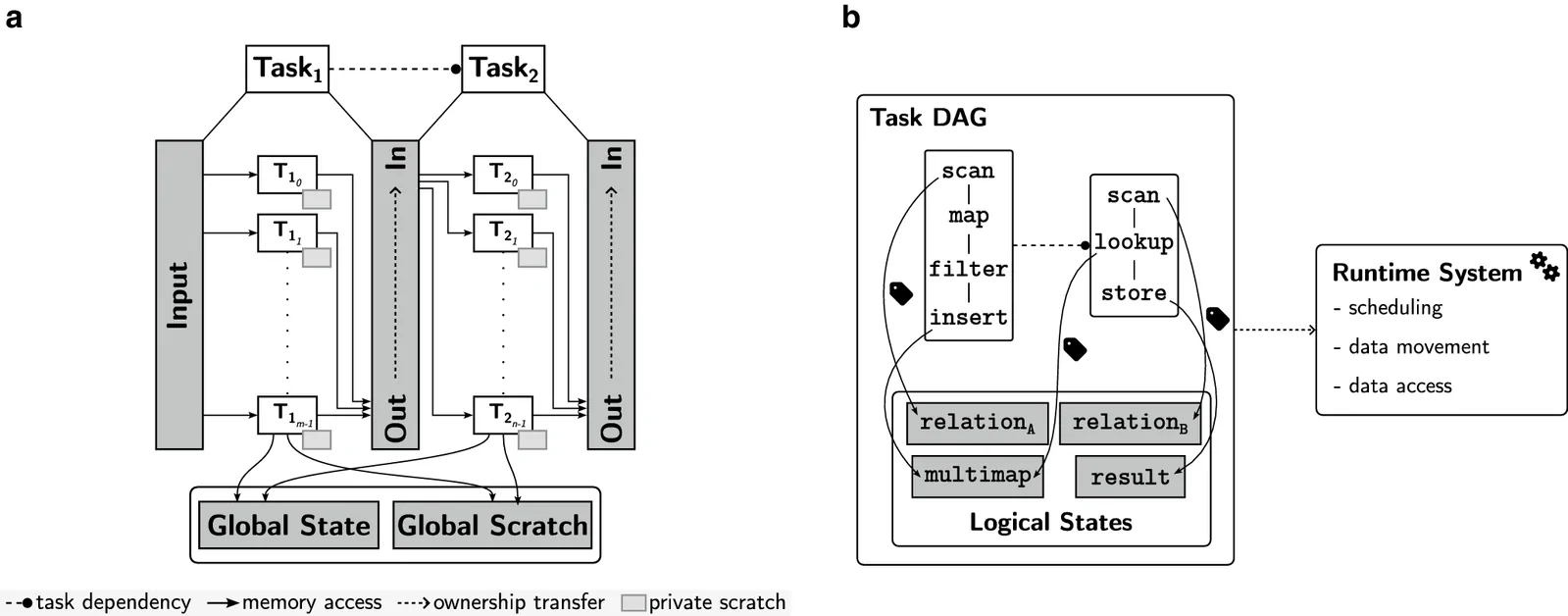
Tasks decompose into per-thread tasklets over *private scratchpad* and interact through shared regions—synchronously via *global state*, asynchronously via *global scratch*. The task DAG, with property-tagged operator-to-state accesses, is fully exposed to the runtime [4, 13]. **a** Tasklets access various memory regions. **b** State accesses are tagged  with properties

The vision sketched by Anneser et al. [4] introduces *logical memory regions* as the abstraction that breaks this coupling. A region is a logical view over physical memory identified by the set of properties it must satisfy relative to the compute device that will use it: latency, bandwidth, persistence, coherence, and access granularity. The same logical region resolves to different physical memory locations depending on the executing device (Fig. 2a): a *Private Scratch* region preferentially maps to DRAM when accessed by a CPU and to GDDR when accessed by a GPU, without any change to application code.

#### A Region Vocabulary

A small set of pre-defined regions captures the memory roles that recur across dataflow systems, each carrying a characteristic property signature (Table 1). Three roles cover the cases: *Private Scratch* for thread-local, non-coherent state; *Global State* for coherent synchronous cross-task synchronization; and *Global Scratch* for coherent asynchronous data exchange between non-adjacent tasks. The same three roles reappear across other applications such as machine learning and streaming pipelines, which is what makes a fixed, small vocabulary of regions generalizable rather than a per-application concern.

**Table 1:** Common Memory Regions

Name	Properties	Purpose
Global State	{coherent, sync}	Syncing tasks
Global Scratch	{coherent, async}	Data exchange
Private Scratch	{noncoherent, sync}	Thread-local data

#### Ownership and Access

Two further mechanisms complete the abstraction. *Memory ownership* attaches each region to a clear owner, whether a task, a job, or the application as a whole. Ownership comes in two modes that translate directly into the memory model the region must provide: a region owned *exclusively* by one task can run with relaxed coherence and weak ordering, whereas one whose ownership is *shared* among concurrent tasks forces the stronger guarantees of cache coherence or strict ordering onto the underlying physical memory. Ownership can also be transferred between successive tasks in a dataflow following the discipline of C*++* move semantics, with the producer’s output region becoming the consumer’s input in place, so that a linear dataflow incurs no copy whenever the underlying physical memory is reachable from both compute devices, and falls back to an explicit copy only when it is not.

*Access interfaces* expose synchronous loads and stores for near memory and an asynchronous interface for far memory, so that a task running against CXL-attached memory, whose latency is comparable to that of a remote NUMA socket, or against a remotely pooled tier, can overlap compute with data movement instead of stalling on the access latency.

Together, these three concepts of regions, ownership, and access modes give the runtime enough information to make placement, scheduling, and lifetime decisions on behalf of the application, without any hardware specifics leaking into the program.

### Workload-Aware Runtime

#### Hardware-Agnostic Operators

The first principle gives a database a declarative interface to memory: regions describe what it needs. The second principle is symmetric on the compute side: the engine exposes its workload to the runtime, namely which tasks run, which states they touch, and what they expect from the hardware, so the runtime can decide where to place state and where to run compute as the underlying hardware changes [13]. A hardware-agnostic engine is the consequence: when a new fabric or accelerator arrives, only the runtime’s mapping policy adapts, not the operators.

#### The State-Centric Task DAG

This exposure takes the form of a *state-centric task DAG* (Fig. 2b): a directed acyclic graph of tasks, each containing a *tasklet* that describes the per-chunk computation, together with a set of explicit *logical states* that the tasks read and write. The DAG’s topology already encodes the producer-consumer dependencies between tasks, which the runtime reads directly. On top of this structure, tasks and their state accesses carry *properties* that record what the topology alone cannot express. These cover both algorithm-specific constraints, such as the requirement that the tasklets of a partitioned hash join consume matching partitions of both inputs, and expectations about hardware, such as low-latency or high-bandwidth access to a particular state, persistence guarantees, or confidentiality. Together, the DAG, the states, and their property annotations give the runtime sufficient structural information to jointly reason about scheduling, state placement, and data movement.

When a new storage technology arrives (e.g., HBM offering more bandwidth, non-volatile RAM offering durability, or a CXL- or NVLink-attached pool offering memory shared across CPU and accelerator), the property annotations already record what each state requires, so adapting the engine is a change to the runtime’s mapping policy rather than to the operators. The same holds for a new interconnect: because the DAG records how each state is accessed, the runtime can weigh moving data against moving compute based on access pattern and operational intensity, exploit cache coherence wherever a fabric provides it, and never need to rewrite the DAG itself. This is the declarative discipline of Sect. 3.1, lifted to the level of whole task graphs.

#### Sub-Operators

A *declarative sub-operator layer* produces the DAG by describing algorithms in terms of explicit accesses to state, so the state nodes in the DAG are the *logical regions* from Sect. 3.1, and the operator-to-state edges carry the access properties the runtime uses for placement.

#### Compilation and Reuse

Layered compilation lowers the sub-operator graph to machine code only after the runtime has placed a tasklet on a concrete device, yielding a CPU or GPU binary as required. Supporting a new accelerator means supplying only the lowest layers of the stack and the lowering passes, which reusable compiler frameworks such as MLIR increasingly provide off-the-shelf. In this way, the operator algebra, the runtime, and the compiler each remain reusable across the technology landscape, and adapting the engine to a new device or fabric becomes a local change instead of a system rewrite.

### Declarative Memory Services

The first two principles give the database declarative interfaces to memory and to compute: regions (Sect. 3.1) describe what each state needs, and the workload DAG (Sect. 3.2) exposes how the engine uses that state. These declarations are written once and hold across fabrics, but satisfying them still requires action: data placement, replication, caching, tiering, and computation offload throughout the underlying infrastructure. The third principle assigns this work to the runtime, which exposes a set of *declarative memory services* that orchestrate execution, much as a buffer manager or an operating-system page cache frees an application from managing physical data placement, making transparent decisions on its behalf [5].

#### A Three-Layer Design

**Fig. 3:**
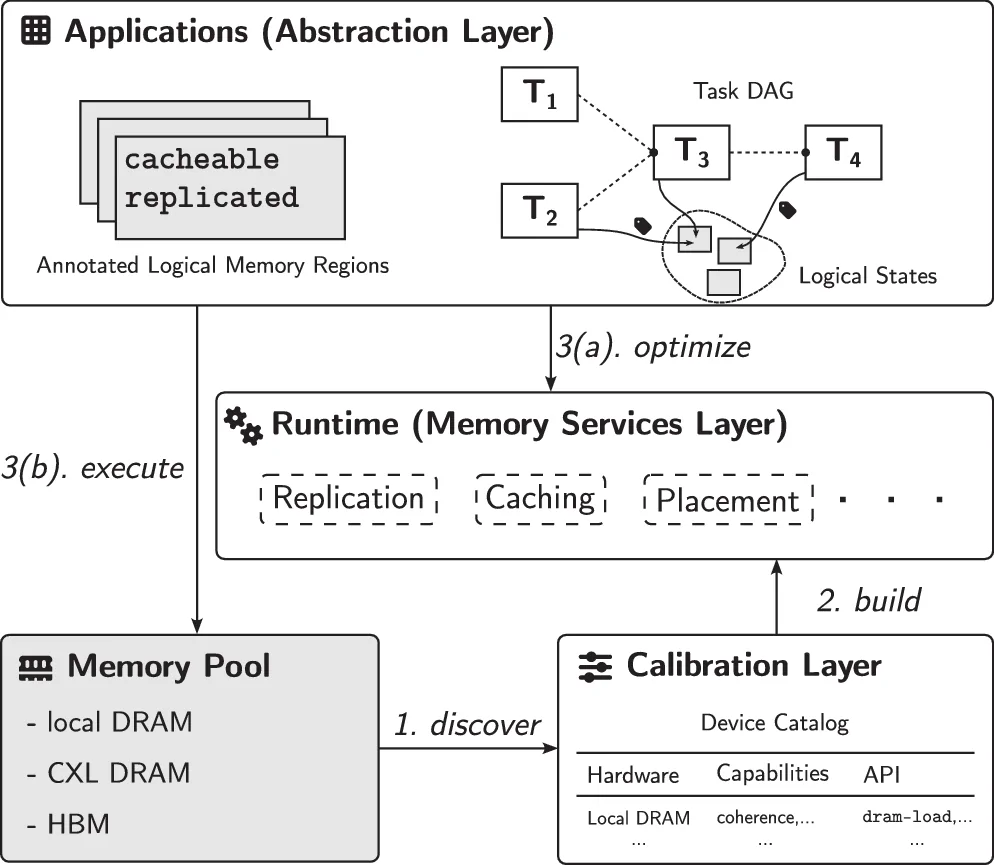
The three-layer structure of declarative memory services [5]. Applications annotate regions and tasks at the top; the runtime composes calibrated, device-specific catalog APIs at the bottom into the services in the middle. Device-specific APIs are never exposed to the application

The proposal is structured as three layers (Fig. 3).

- An *abstraction layer* lets applications annotate logical regions and dataflow tasks with properties drawn from a common taxonomy, covering both quantitative performance bounds (e.g., latency and bandwidth targets) and qualitative attributes (e.g., volatility, granularity, coherence, fault semantics).
- A *calibration layer* maintains a device catalog that discovers each available memory device, records its capabilities, and exposes device-specific APIs such as *dram-load* and *cxl-load* for the runtime’s internal use only; these APIs are never exposed to the application.
- Between them, a *memory services layer* consumes those APIs, implementing common services such as caching, replication, data placement, and SLO monitoring by composing them.

Each such service can be realized in several ways, for instance, offloading via a memory-side compute pool, via a PIM unit, or not at all, and the runtime selects an implementation based on application annotations and the current device mix.

#### Two Styles of Declaration

The abstraction layer admits two styles of declaration, and an application may use either or both. At the fine grain, individual logical regions are annotated at the block level (e.g., a B^+^-tree node declared *[cacheable, latency **<** 10 **μ**s]*), which suits low-level data structures. At the coarse-grained level, an application is expressed as a dataflow graph whose tasks carry the same properties as service-level objectives, which suits end-to-end pipelines assembled from operators.

#### Resolving to Hardware

The calibration layer grounds these declarations in hardware by recording, per device, both a capability profile (local DRAM is coherent and byte-addressable, CXL-attached DRAM only partially coherent) and the concrete APIs that realize it. These APIs play the role that physical operators play in a query engine: choosing between a CXL-based and an NVLink-based realization of the same access is the memory-layer analog of choosing between a hash join and a sort–merge join, and it is the memory-services layer that makes the choice.

#### Case Studies

A disaggregated B^+^-tree on RDMA primitives must hard-code its caching, partitioning, and offloading strategies; under declarative memory services [5], the same tree is written once with nodes annotated *cacheable* and operations *offloadable*. Similarly, a tiered buffer manager can be told that scan-once pages should bypass the cache, hash-table pages must remain coherent, and cold pages may be re-encoded en route to a remote tier, hints OS-level tiering services cannot exploit. In every case, the same application code retargets to RDMA, CXL, or PIM as the underlying hardware changes; the device-specific decisions move into the services and out of the application.

### Ongoing Work

Each principle is the subject of active follow-up work in our group, across three threads that translate the declarations of the previous subsections into operational systems.

#### Region-Aware Engine Design

We are building a shared-memory-native analytical query engine that can distribute data across heterogeneous hardware environments. Our research suggests that adapting existing, local-memory-native engines to shared environments is prohibitively complex. In a shared memory environment, the standard abstraction of a pointer breaks down. This means many of the usual programming language facilities—dynamic dispatch, function pointers, standard library shared pointers and containers—cannot be used without special care.

Instead, we argue that the system’s architecture must be designed with heterogeneous memory in mind from the very conception. The system needs to share the state of the execution pipelines coherently, while read-only structures can be put in non-coherent pools. We distinguish these on the type system level with different *kinds* of pointers that are explicitly not interchangeable. Preliminary results show little overhead compared to local in-memory parallel processing and distribution via shared-memory files. More rigorous experiments on real CXL hardware are still ongoing.

#### Workload-Driven Placement

A second thread builds a placement policy that exploits the state-centric design of Sect. 3.2: because the annotated task DAG is exposed to the runtime, placement and migration decisions can be made based on states and their declared access patterns rather than opaque pages.

A precedent for this style of policy already exists at the memory–storage boundary: prior work [15] hides NVMe spilling from operators behind a partition-aware interface, using runtime metrics to silently materialize partitions to storage and stream them back on demand. As the line between memory and storage blurs—CXL-attached memory now sits closer to fast SSDs than to local DRAM—the same approach transfers naturally to the boundary between memory tiers, with state-level annotations standing in for partition-level metrics.

Each state in our prototype carries declarative access properties—for instance, random vs. sequential, read-only vs. append-only—which the runtime combines with observed metrics to migrate the state between local DRAM and CXL-attached memory. A state built under random access (e.g. a hash-aggregation result) is demoted to the higher-bandwidth but more distant tier once a downstream operator begins reading it sequentially (e.g. a final pass over the aggregation result), without the operator observing the move.

Unlike a traditional buffer manager that relies on general-purpose replacement policies such as LRU, our policy operates at state granularity and exploits its *declared* access properties. LRU and its variants see only opaque pages and infer behavior solely from *observed* accesses. Measurements on join- and aggregation-heavy workloads are encouraging, and we are now extending the prototype into a live runtime that promotes and demotes states as the DAG executes.

#### Cross-Stack Runtime

A third thread builds the runtime side of the design model, where the declarative interfaces of the previous two principles are resolved against the underlying hardware. A single runtime must reconcile two streams of annotations: region properties surfaced at the bottom of the engine (latency, coherence, persistence per allocation), and sub-operator properties surfaced at the top (access patterns, operator constraints per state), so that each state’s location respects both how it is held and how it will be used.

Our aim is to build a *region-typed allocator* that spans CXL-attached memory and fast NVMe, treating the two as a single graded tier rather than separate memory and storage layers. The allocator reuses the engine’s existing region and access-pattern annotations, making the classical buffer manager and the OS page cache local-versus-remote special cases of a more general placement policy. On top of the allocator, the runtime owns the physical placement of state—migrating hot to closer tiers, demoting cold, and replicating across devices when a persistence or fault property requires it—driven by annotations rather than access traces alone. A further responsibility on the same runtime is mediating per-region SLOs across tenants on shared resources, detecting violations, and re-binding regions to different service implementations.

We treat these threads as initial validations of the design, and the experience they generate shapes the open agenda discussed in Sect. 5.

## Related Work

### Pre-CXL Disaggregation

RDMA-tuned tree indexes [31], far-memory data structures [2], and POLARIS’s Cell abstraction [1] represent the pre-CXL state of the art on non-uniform and remote-memory access, with POLARIS already making state placement a runtime decision rather than a query-plan one. LegoOS [24] reorganized the kernel around the disaggregation principle.

### Partitioned Global Address Space

The HPC community has long worked with a partitioned-but-globally-addressable model in which languages such as Chapel expose locality as a first-class property of data [6]; the principles of Sect. 3 can be read as its database analogue, with regions and ownership playing the role of PGAS locales.

### CXL Foundations, Measurements, and Systems

The CXL standard is documented from version 1.0 through 3.0 [25], and a growing body of measurement studies on real hardware [3, 21, 26] confirms and quantifies its latency and bandwidth envelope. Vistara reports the first hyperscale CXL deployment, with tail latencies comparable to local DRAM [10]. On the systems side, Farview [14] offloads operators to disaggregated memory; Pasha [12] and Tigon [11] target CXL pods directly; CTXNL co-designs hardware and software for transactions on CXL [28].

### Pooling, Topology, and Tiering

Pond first measured cloud-scale CXL pooling [20]; further work questions whether pods must be flat. Octopus proposes sparse, multi-ported-device topologies organized into islands [30], a HotOS paper argues that a CXL pool can subsume PCIe disaggregation [29], and a HotNets counter-argument pushes back on pooling economics at small scales [19]. At the OS level, work involves transparent page placement [22] and dynamic page classification [16].

### Vision and Position Papers

The vision for memory-centric databases was set out at CIDR [8]; the principles of Sect. 3 are drawn from our prior work [4, 5, 13]. Beyond our own line, others argue that CXL revives scale-up architectures [18]; a parallel thread argues that RDMA-enabled disaggregation makes distributed shared-memory databases viable again [27].

### Position Relative to Prior Work

The works above characterize a specific technology or build an engine that exploits one of its features; the principles of Sect. 3 differ in kind. Logical memory regions abstract over the device-specific placement that OS tiering hides from applications; annotated state-centric task DAGs parameterize the algebra that most engines hardwire to a single memory shape; declarative memory services lift caching, replication, and offload policies that CXL-specific database systems bake into the engine.

## Outlook

Disaggregated memory is arriving as several technologies, and we argue that database architectures should be memory-centric, letting the engine declare its memory needs while a runtime resolves those declarations against available hardware. Several open challenges remain.

### Programming Abstractions

These principles prescribe a vocabulary of properties but not the language in which to express them: systems languages such as C*++* or Rust preserve performance yet resist runtime reasoning and rewriting, whereas higher-level declarative languages aid optimization but raise sandboxing, compilation, and adoption questions [13].

### Scheduling and Placement

Once properties are resolved to hardware, the runtime must schedule compute and place state across memory with different characteristics. This goes beyond NUMA-aware techniques [17] and the opaque tiering of existing OS work [16], neither of which can take application-level hints.

### Failure Semantics and Isolation

Memory pools introduce failure modes the process-local model ignores (e.g., link loss in a switched pool, partial faults in a switchless one [30]). Expressing them as declarative properties is straightforward; the underlying replication and checkpointing mechanisms, and their interactions with transaction protocols, are not. Beyond failure, a pooled fabric is inherently multi-tenant, raising the performance and security-isolation problems hyperscalers face but with the tenant boundary now a logical region, not a process address space.

We believe that the next decade of database research will be productive in proportion to the abstractions it builds, not the technologies it bets on. Memory has become the organizing principle of data centers, and the engines that survive the resulting hardware shifts will be the ones whose internal structure is organized the same way.

## Data Availability

No datasets were generated or analysed during the current study, as this article presents a conceptual and architectural discussion.
